# Research Using Social Media to Recruit Research Participants Should Proceed With Caution. Comment on “Telemanagement of Home-Isolated COVID-19 Patients Using Oxygen Therapy With Noninvasive Positive Pressure Ventilation and Physical Therapy Techniques: Randomized Clinical Trial”

**DOI:** 10.2196/34437

**Published:** 2022-05-02

**Authors:** Chih-Wei Chen, James Cheng-Chung Wei

**Affiliations:** 1 Institute of Medicine Chung Shan Medical University Taichung Taiwan; 2 Department of Allergy, Immunology & Rheumatology Chung Shan Medical University Hospital Taichung Taiwan; 3 Graduate Institute of Integrated Medicine China Medical University Taichung Taiwan

**Keywords:** telemedicine, oxygen therapy, noninvasive positive airway pressure, BiPAP, osteopathic medicine, physical therapy, SARS-CoV-2, COVID-19, teletherapy, telemanagement

We read with great interest the research article by Adly et al [1] regarding the nonpharmacological respiratory treatment methods for home-isolated patients with COVID-19, involving the use of a newly developed telemanagement health care system. We appreciate the authors’ valuable contribution to providing a deeper understanding of the effectiveness of home-based oxygen therapy with bilevel positive airway pressure (BiPAP) and that of osteopathic manipulative respiratory and physical therapy techniques in impeding exacerbation of early-stage COVID-19 pneumonia. However, the study should be further discussed in terms of research design, patient recruitment technologies, and the influence of the telemanagement system on health care development.

To begin with, the researchers randomly recruited 60 patients for their study through social media by using a snowball subject recruitment technique. However, the health conditions of these patients (eg, age and underlying co-morbidities), which significantly affect their outcomes [2], were not considered in the analysis. Moreover, the eligibility of recruited patients has not been well addressed in their publication. The sample size and power calculation should also be presented in a clinical trial protocol.

Moreover, the authors used the snowball subject recruitment technique through social media to recruit patients, which was completely random and could not ensure that all patients met the eligibility criteria. However, successful patient recruitment requires a rational clinical design, efficient patient identification and randomization, which can be fulfilled by various information technologies such as data mining, artificial intelligence, and automated alerts [3]. Furthermore, the echo chamber effect on social media could also result in the limitation of patient recruitment [4]. Hence, we suggest that the authors use suitable technologies based on a comprehensive database for patient recruitment.

Furthermore, the national health care database has been increasingly developed and used as a comprehensive database for clinical trials [5]. The employment of the telemanagement system in home-based treatment for patients with COVID-19 allows for data collection through the system and uses fewer human resources. The integration of the national health care database and the telemanagement system could thus allow health care workers to provide long-distance health care not only in the context of COVID-19 treatment but also in health care in the future society. Hence, the authors are suggested to discuss the influence of the implementation of telemanagement together with the national health care database on health care development.

Above all, this study contributes to providing a deeper understanding of the effectiveness of home-based oxygen therapy with BiPAP, and that of the osteopathic manipulative respiratory and physical therapy techniques in impeding exacerbation of early-stage COVID-19 pneumonia. Further studies would likely enhance the research design, implement patient recruitment by more suitable technologies, and discuss the influence of the telemanagement system on health care development.

## Abbreviations

BiPAP: bilevel positive airway pressure
